# Influence of Initiator Content and Polymerization Conditions on the Properties of Polyacrylate Mortar

**DOI:** 10.3390/ma18204737

**Published:** 2025-10-16

**Authors:** Zhengqiang Huang, Chong Han, Tianhang Zhang, Dongyang Guo, Yonggui Dai, Wencheng Ding

**Affiliations:** 1 School of Water Conservancy and Transportation, Zhengzhou University, Zhengzhou 450001, China; hzq124@gmail.com (Z.H.); hanchong@gs.zzu.edu.cn (C.H.); 17538132879@163.com (D.G.); daiyg@gs.zzu.edu.cn (Y.D.); dwc4113@gs.zzu.edu.cn (W.D.); 2 Zhongyuan Institute, Zhejiang University, Zhengzhou 450000, China

**Keywords:** polymer, mortar, BMA, BPO, DMA, microstructure

## Abstract

An experimental investigation was conducted to study the effect of initiator content and polymerization temperature on the mechanical and bonding properties of polyacrylate mortar. Initiator content was controlled in 0.1, 0.2, 0.3, 0.4, 0.5, 0.6, 0.7, 0.8, 0.9 and 1.0% and polymerization temperature was set at −20, 0, 20, 40, and 60 °C in aggregation process, respectively. The mixture of butyl methacrylate (BMA), benzoyl peroxide (BPO) and N, N-dimethylaniline (DMA) was added to the aggregate composed of quartz sand and silica fume (SF) according to the ratio of monomer to aggregate of 1:4. Results showed that compressive, flexural, tensile, and bonding strengths of polyacrylate mortar decreased with increasing temperature but increased with higher initiator content. The optimal initiator content was 0.6%. Although the highest strength was observed at −20 °C, this curing condition is not easy to achieve in practice and should be considered as laboratory optimization. According to the room temperature, 20 °C can be selected as the best polymerization temperature. SEM observations indicated that the polyacrylate cementitious material cross-linked to form a film, with a dense polymer distribution at the interface that improved interfacial continuity. These findings provide mechanistic insight for optimizing initiator content and curing conditions to enhance the mechanical and bonding performance of polyacrylate-based cementitious composites.

## 1. Introduction

The construction of major infrastructures such as super high-rise buildings and long-span bridges, as well as the repair and reinforcement of existing structures, imposes higher requirements on the performance of building materials. While traditional cement-based materials offer advantages of low cost, high strength, and adaptability [1,2], they also suffer from inherent limitations throughout their service life. These include excessive carbon emissions, intrinsic brittleness, and low tensile strength [3,4,5], which have become major obstacles to sustainable development in the construction industry. Consequently, the development of novel, high-performance, low-carbon binder systems has become an urgent priority in civil engineering.

In this regard, the incorporation of polymers provides a promising solution [6]. Their application in civil engineering materials is generally categorized into three forms: polymer-modified concrete (PMC), polymer-impregnated concrete (PIC), and polymer concrete (PC). PMC is produced by introducing polymer emulsions or powders into conventional cementitious systems and is mainly used in repair, coatings, and waterproofing [7,8]. PC, in contrast, is manufactured by combining well-graded aggregates or fillers with polymer resins that partially or completely replace cement binders [9,10]. Unlike conventional cement concrete, the properties of PC and PMC are strongly influenced by polymer type, aggregate, and curing conditions [11,12]. PC is particularly attractive because of its simple preparation, rapid hardening, abrasion resistance, and high strength [13,14]. Experimental studies have shown that its compressive strength is two to three times higher than that of ordinary concrete, while its flexural and tensile strengths can be up to five times greater [14]. These improvements arise from the polymer binder, which enhances adhesion and strength when combined with aggregates such as sand or gravel [15]. For example, Heidarnezhad [16] demonstrated that higher polymer dosages significantly increase compressive, splitting tensile, and flexural strengths, with further improvements observed at lower curing temperatures. Lópe et al. [17] achieved compressive strengths of 80 MPa using unsaturated polyester resin with silica sand, while Zeldin et al. [18] synthesized homogeneous PC via copolymerization of acrylonitrile, acrylamide, and trimethylolpropane triacrylate, producing compressive strengths up to 183 MPa alongside excellent chemical stability and corrosion resistance. Moreover, the rapid curing of PC significantly shortens project timelines, translating into both environmental and economic benefits [19].

Among available polymer systems, polyacrylates have attracted particular attention due to their unique molecular structures and superior durability. Compared with common alternatives such as epoxy resins [20] and polyurethanes [21], polyacrylates offer outstanding weather resistance, UV stability, low shrinkage, and strong aggregate wettability, enabling more reliable long-term performance [8]. Their excellent flexibility and adhesion also enhance the compactness of the interfacial transition zone (ITZ) [22], strengthening the bond between mortar and aggregates while mitigating microcrack initiation and propagation. These advantages make polyacrylate mortars a promising candidate for advanced binder systems and future high-performance materials. Silica fume (SF), widely used as a mineral admixture in cementitious materials, also plays a crucial role owing to its high strength, large surface area, and pozzolanic activity [23,24,25]. Its inclusion reduces porosity and enhances mechanical performance [26,27], and in fiber-reinforced concrete it improves early bonding between fibers and paste [25]. When combined with polymers, SF often exhibits strong synergistic effects, yielding superior performance. For instance, the addition of styrene–acrylic copolymers and SF significantly improves the tensile bond strength of mortars [28].

Despite extensive research on the influence of polymer type and dosage on strength development, one critical distinction between PMC and PC remains underexplored. In PMC, the cementitious phase dominates, potentially masking the effects of polymerization parameters. In PC, however, the fully polymerized matrix means that even minor changes in synthesis conditions, such as initiator content or curing temperature, can profoundly affect the polymer network and, in turn, the macroscopic material properties. Initiators, as essential components of radical polymerization, directly control radical generation, influencing molecular weight, crosslinking density, and network integrity. Likewise, curing conditions are equally decisive. Temperature not only dictates initiator decomposition kinetics but also affects monomer mobility and reactivity, thereby determining the rate and uniformity of network formation. Insufficient temperatures may hinder curing, while excessive ones may trigger side reactions and structural heterogeneity, undermining performance. Although the importance of these factors is well recognized, systematic studies on the combined effects of initiator content and polymerization temperature on polyacrylate mortars remain scarce, particularly under broad temperature ranges including subzero conditions, which are rarely addressed in the recent literature. This study therefore aims to systematically investigate the influence of initiator content and curing temperature on the mechanical and bonding performance of polyacrylate mortars. The results are expected to fill this research gap, provide a theoretical basis for their application in cold environments, and offer mechanistic insights to guide the design of advanced, high-performance cement-based composites.

## 2. Materials and Methods

### 2.1. Materials

In this study, chemically pure butyl methacrylate produced by Jinan Yuanxiang Chemical Co., Ltd. (Jinan, China). was used as the cementitious material. Silica fume (SF), supplied by Shanghai Meibao New Materials Co., Ltd., (Shanghai, China), had a specific surface area of 20.0 m^2^/g and an average diameter of 0.15 μm. Quartz sand and silica fume (SF) were used as fine aggregates. The physical properties of various raw materials are shown in Table 1. The composition of the aggregate is shown in Table 2.

**Figure 1:**
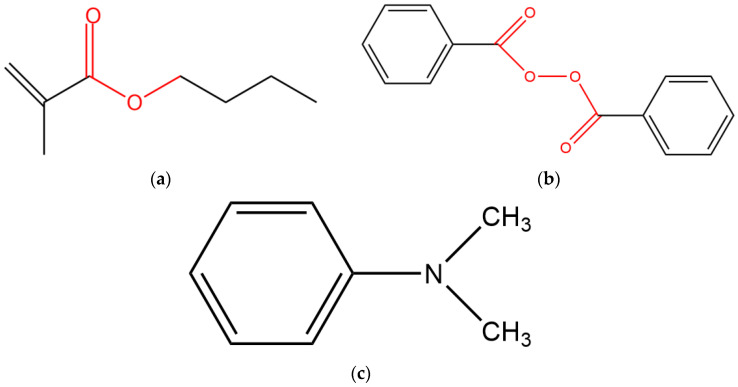
Structural formula of the selected monomer and initiator (**a**) BMA (**b**) BPO (**c**) DMA.

Since the polymerization temperature investigated in this study involves −20 °C, a low-temperature initiation system must be employed to achieve polymerization at such low temperatures. In this study, an oxidation-reduction reaction system composed of benzoyl peroxide (BPO) and N,N-dimethylaniline (DMA), both produced by Shandong Guohua Chemical Co., Ltd. (Heze, China), was utilized. BPO decomposition rapidly initiates monomer polymerization with high-quality polymer output [29,30]. Compared to peroxide initiators, the inclusion of DMA as a promoter primarily accelerates peroxide decomposition, enabling polymerization initiation within a short timeframe even at ambient or low temperatures [31,32]. The structural formulas of the monomer and initiator are shown in Figure 1. Tap water was used in all experiments.

### 2.2. Mix Proportion and Mixing Procedure

In this study, the fixed mix proportion of polyacrylate mortar was set as follows: butyl methacrylate (BMA) was used as the cementitious material, with quartz sand and silica fume (SF) as aggregates, at a mass ratio of 1:4. The mixing procedure was carried out as follows: first, quartz sand and SF were premixed for 1 min to achieve uniform dispersion. Then, the BMA monomer, initiator benzoyl peroxide (BPO), and accelerator N,N-dimethylaniline (DMA) (mass ratio 0.53:1) were added, followed by mixing for an additional 3 min, giving a total mixing time of 4 min. The fresh mortar was then cast into the corresponding molds for testing.

To prevent monomer volatilization and moisture loss, all specimens were sealed before being placed into temperature-controlled equipment. The specific steps were as follows: (1) the freshly cast molds were quickly placed into self-sealing bags; (2) most of the air inside the bags was expelled, and the bags were tightly sealed; (3) the sealed bags were then placed in a constant-temperature chamber. This method effectively ensured that the polymerization reaction proceeded in a closed environment and maintained the stability of the mix proportion.

### 2.3. Experimental Program and Testing Methods

#### 2.3.1. Polymerization Time Experiment

To determine a unified and sufficient curing time standard, polymerization kinetics experiments were first conducted. Mortar specimens containing 1% initiator (BPO/DMA) were prepared and cured at 60 °C. The curing times were set at 0, 1, 2, 3, 4, 5, 6, 12, and 24 h. The purpose of this experiment was to establish the minimum curing time required to ensure that the polymerization reaction was essentially complete, serving as a reference for all subsequent experiments in this study.

#### 2.3.2. Low-Temperature Polymerization Test

In this study, −20 °C was selected as the low-temperature polymerization condition. Its feasibility is based on the unique characteristics of the BPO/DMA redox initiation system. This system generates free radicals through electron transfer reactions, with an extremely low activation energy, which allows it to maintain high reactivity even at low temperatures.

To ensure the rigor of the low-temperature experiments, all specimens were immediately placed in a low-temperature freezer after casting. The internal temperature was continuously monitored and recorded to ensure that fluctuations remained within an acceptable range. It should be noted that the freezing point of BMA monomer is approximately −76 °C; thus, it remains in liquid form at −20 °C, ensuring adequate fluidity and sufficient contact between the monomer and initiator, thereby guaranteeing the feasibility of the reaction. All specimens were cured in a sealed environment to prevent moisture loss and monomer volatilization.

#### 2.3.3. Mechanical Tests

**Figure 2:**
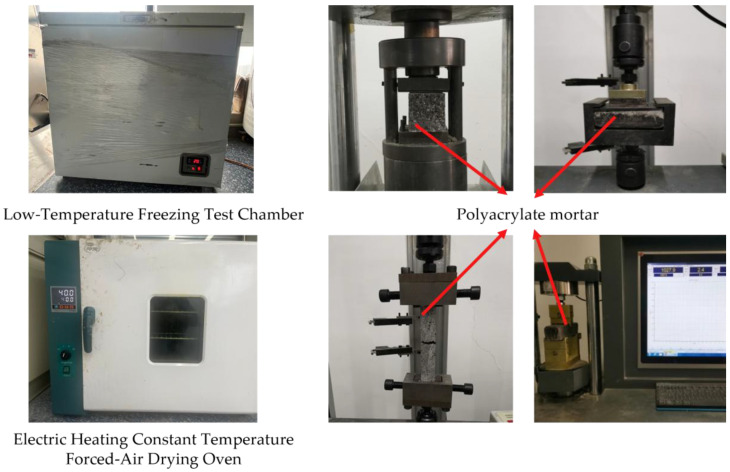
Testing methods.

After the specimens completed curing under the designated conditions, they were demolded. The compressive strength, tensile strength, and bond strength of the specimens were tested in accordance with the standards GB/T 17671-2021 [33], JTG E50-2006 [34], and JGJ/T 70-2009 [35], respectively. The test method is shown in Figure 2. To ensure the comparability of mechanical property test results and the reliability of conclusions, all test data underwent systematic statistical analysis. Each experimental condition group included three independent parallel samples (*n* = 3), with data presented as mean ± standard deviation. Homogeneity of variance tests and significance tests were conducted concurrently. Levene’s test assessed variance homogeneity among different groups, while analysis of variance (ANOVA) determined whether the overall effects of different experimental conditions on mechanical properties were statistically significant. Additional experimental data are provided in the Supplementary Materials Table S1.

#### 2.3.4. SEM Analysis

To elucidate the microscopic mechanisms underlying changes in macroscopic performance, scanning electron microscopy (SEM) was employed to observe the fracture morphology and interfacial structure of selected specimens. The samples were dried, gold-sputtered, and then examined under a specified accelerating voltage. The analysis focused on features such as the continuity of the polymer film, the bonding interface with aggregates, and the presence of micro-defects.

## 3. Results and Discussion

### 3.1. Polymerization Time

**Figure 3:**
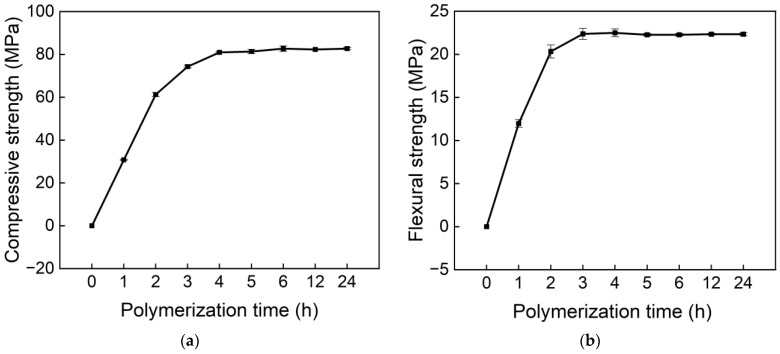
(**a**) Compressive strength vs. polymerization time; (**b**) Flexural strength vs. polymerization time.

In this study, to investigate the effects of polymerization temperature and initiator content on material properties, it was necessary to control polymerization time as a specific variable. To this end, specimens prepared with 1% initiator in BMA were cured in an oven at 60 °C for different polymerization durations. The compressive strength and flexural strength of the specimens at various curing times were tested and plotted, as shown in Figure 3. Additionally, Levene’s test for homogeneity of variances indicated that both compressive strength and flexural strength (*p* > 0.05) satisfied the assumption of homogeneity of variances. Univariate analysis of variance revealed that polymerization time exerted an extremely significant effect on both compressive strength and flexural strength (*p* < 0.05).

Within the first 3 h, both compressive and flexural strengths increased significantly with curing time. For specimens with curing times longer than 3 h, only slight changes in compressive strength were observed. A possible explanation is that the decomposition temperature of the initiator is lower than the polymerization temperature (60 °C), allowing monomer polymerization to essentially complete within approximately 3 h [22].

This study also involves polymerization at −20 °C and, taking into account relevant findings on the influence of temperature on polymerization, a curing time of 12 h was adopted as the standard polymerization duration for this investigation. It should be noted that this 12 h curing time was selected for consistency in the experiments and does not necessarily represent the optimal polymerization time.

### 3.2. Initiator Content and Polymerization Temperature

**Figure 4:**
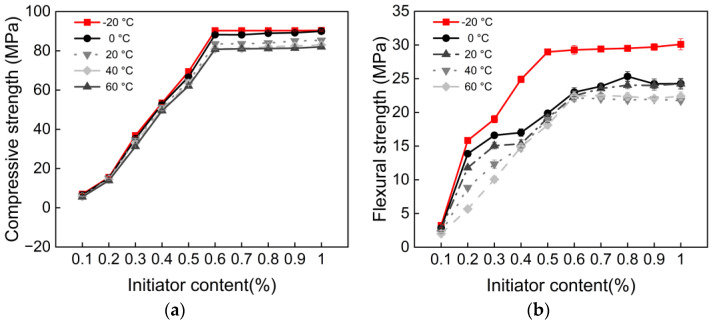
(**a**) Compressive strength vs. initiator content; (**b**) Flexural strength vs. initiator content.

**Figure 5:**
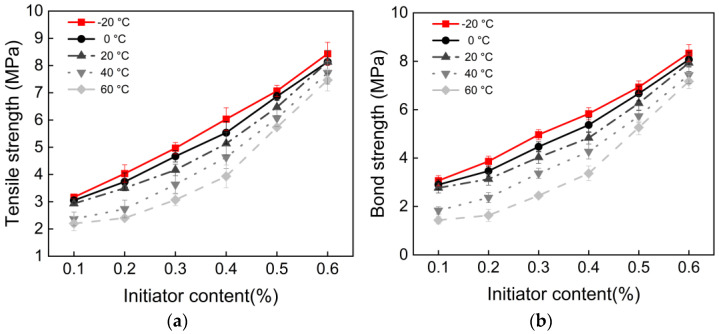
(**a**) Tensile strength vs. initiator content; (**b**) Bond strength vs. initiator content.

To achieve better polymerization results, the compressive strength and flexural strength of samples were evaluated by varying the initiator content and polymerization temperature (−20, 0, 20, 40, and 60 °C). The compressive and flexural strengths of the mortar increased with higher initiator content, as shown in Figure 4. The effects of initiator content and polymerization temperature on the mechanical properties of the material were evaluated using Levene’s test and two-way ANOVA. Levene’s test results indicated that compressive strength, flexural strength, tensile strength, and bond strength all satisfied the assumption of homogeneity of variance (*p* > 0.05). Two-way ANOVA revealed that both initiator content and polymerization temperature exerted highly significant main effects (*p* < 0.05) on compressive, flexural, tensile, and bond strengths. At a polymerization temperature of −20 °C and an initiator content of 0.6%, compressive strength increased by 83.3 MPa compared to 0.1%, and flexural strength increased by 25.7 MPa. Beyond 0.6%, strength stabilized at 90 MPa. At a polymerization temperature of 60 °C, compressive strength, flexural strength, and bond strength de-creased by 10.5%, 23.3%, and 15%, respectively, compared to −20 °C. Based on compressive and flexural strength results, the most significant range of initiator content variation was selected. Figure 5 illustrate the relationship between tensile strength, bond strength, and initiator content, showing nearly identical trends for both strengths as initiator content increases. At a polymerization temperature of −20 °C and an initiator dosage of 0.6%, the tensile strength of the polyacrylate mortar increased by 5.45 MPa compared to the 0.1% dosage. The bond strength of the polyacrylate mortar at 0.6% initiator content increased by 5.28 MPa compared to the 0.1% dosage.

The core function of initiator content lies in regulating radical concentration and polymerization kinetics. As shown in Figure 4 and Figure 5, when initiator content increased from 0.1% to 0.6%, all strength indices rose rapidly. This can be attributed to the increased generation rate and concentration of primary radicals, which enhanced monomer conversion and promoted the formation of a highly crosslinked three-dimensional polymer network with higher molecular weight [9]. However, once the initiator content exceeded the critical threshold of 0.6%, the growth in strength is stabilizing. This indicates that monomer conversion was essentially complete, and excess radicals no longer contributed effectively to network formation. Instead, they were consumed through termination reactions such as radical recombination, potentially broadening the molecular weight distribution and limiting further optimization of the network structure.

**Figure 6:**
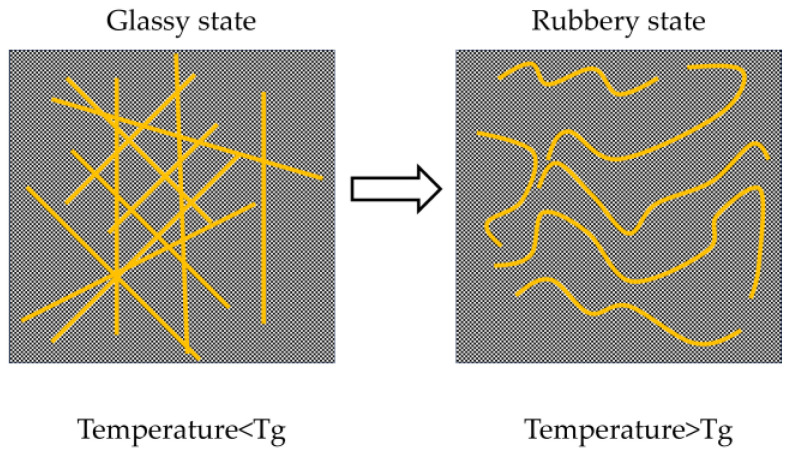
Diagram of Temperature Effects on Polymer Chains.

It is also noteworthy that all strength properties showed a negative correlation with temperature. This can be explained by polymer chain dynamics and network stability. As shown in Figure 6. The glass transition temperature (T_g_) of PBMA is approximately 18 °C, and its flow temperature is around 120 °C. At sub-T_g_ temperatures (e.g., −20 °C), polymer chains in the mortar are in a glassy state, with severely restricted segmental mobility. In this state, the polymer network is rigid, resulting in high compressive and tensile strength [9]. When the temperature rises to around 20 °C, the polymer enters a rubbery or highly elastic state, where increased chain mobility facilitates deformation under applied stress, thereby reducing stiffness and strength.

In addition, temperature influences radical stability during in situ polymerization. At 60 °C, the BPO/DMA redox reaction proceeds too rapidly, generating excessively high radical concentrations in a short time. This accelerates chain termination, leading to more branched structures or low-molecular-weight polymers, ultimately lowering the quality and uniformity of the crosslinked network and reducing strength. Furthermore, thermal softening near T_g_ weakens cohesive interactions both within the polymer matrix and at the polymer–cement interface, further decreasing compressive, flexural, and tensile strength. Therefore, the interplay among chain mobility, radical kinetics, and thermal softening across the glass-to-rubber transition range provides a comprehensive mechanistic explanation for the temperature-sensitive behavior observed in PBMA cement mortar [36,37].

**Figure 7:**
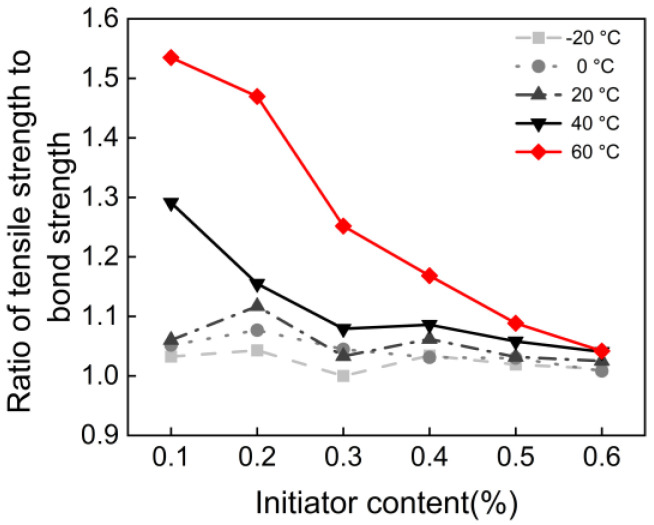
Ratio of tensile strength to bond strength vs. initiator content.

**Figure 8:**
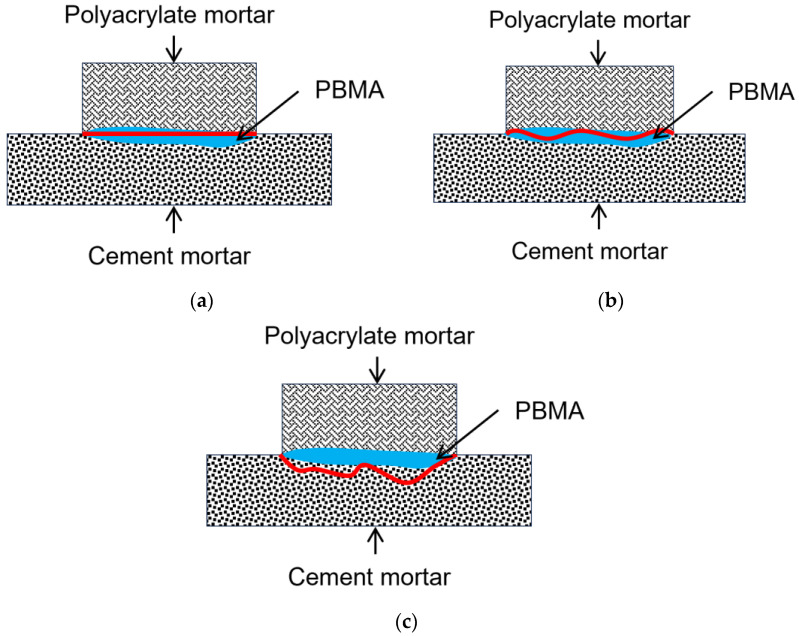
Failure modes of specimens obtained from tensile test: (**a**) failure occurs across the interface; (**b**) interfacial failure accompanied with some repair mortar or substrate concrete fracture; (**c**) failure occurs in the substrate concrete. The red line indicates the interface where the test specimen failed.

To further clarify the failure behavior of polyacrylate mortar, the relationship between tensile strength, bonding strength, and failure mode was analyzed, as shown in Figure 7 and Figure 8. The ratio of tensile to bonding strength is a key parameter for predicting the failure mode of bonded joints. Figure 7 shows that this ratio slightly exceeds 1 under all conditions. This indicated that the interfacial phase is the weakest link and failure was more likely to occur at the interface. With increasing initiator content and decreasing temperature, the ratio gradually approached 1, stabilizing around 1.05 at 0.6% initiator content across all temperatures.

Figure 8 illustrates the observed failure modes, which can be classified into three categories: (1) interfacial failure across the interface (denoted as “a”); (2) mixed failure involving partial fracture of the repair mortar or substrate concrete (denoted as “b”); and (3) cohesive failure within the substrate concrete (denoted as “c”). The results revealed a clear transition in failure behavior: with increasing initiator content, the failure mode shifted from interfacial failure to mixed and cohesive failure. At lower initiator contents, most specimens failed either at the interface or within the substrate concrete. This suggests that higher crosslinking enhances both bulk and interface mechanical performance, favoring cohesive or mixed-mode failure under load. Cohesive failure is generally preferable as it indicates bond strength reaching material limits.

### 3.3. SEM Observations

To investigate the effect of initiator content on the microstructure of polyacrylate mortar, this study selected two representative initiator contents, 0.1% and 0.6%, for scanning electron microscopy (SEM) observation. All specimens were cut at a depth of 2–3 mm from the surface, vacuum-dried at 40 °C for 24 h, gold-sputtered, and then observed using a Hitachi SU8010 SEM (Hitachi High-Tech Corporation, Tokyo, Japan) at an accelerating voltage of 15 kV.

**Figure 9:**
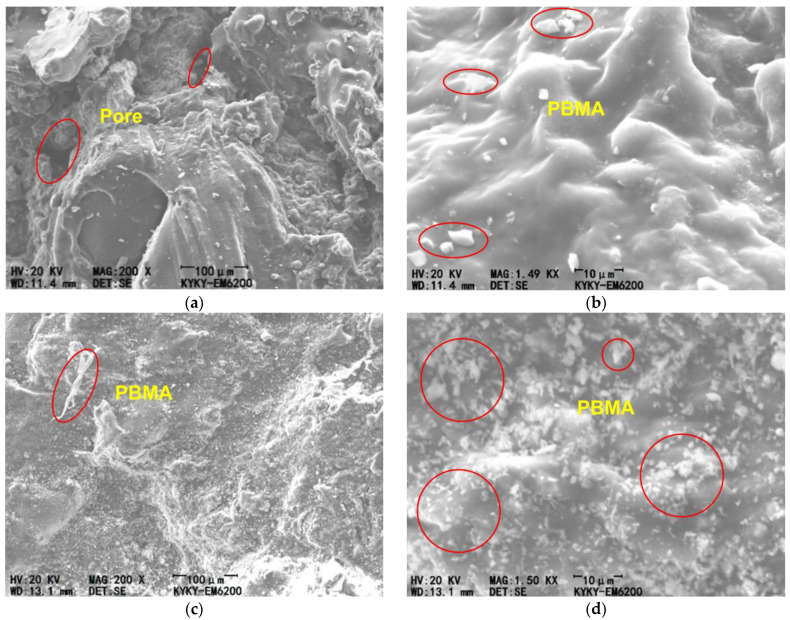
(**a**,**b**) SEM observation of polyacrylate mortar with 0.1% initiator content; (**c**,**d**) SEM observation of polyacrylate mortar with 0.6% initiator content.

The observations revealed that initiator content significantly influences the morphology and distribution of the polymer phase. In the 0.1% sample (Figure 9a,b), the polymer phase exhibited a discontinuous distribution, with obvious microcracks and pores visible in the interfacial region, along with partial separation between the polymer and aggregates. Such structural features may lead to stress concentration, which is consistent with the lower mechanical strength observed in macroscopic tests.

In contrast, the 0.6% sample (Figure 9c,d) displayed a more continuous and compact polymer network. The polymer phase fully encapsulated the aggregate particles, with indistinguishable interfacial transition zones, forming a dense composite system. This uniform microstructure provides excellent mechanical performance, explaining the higher strength observed in macroscopic tests. Figure 9 show the presence of numerous “white particles” distributed along the bonding interface. At a dosage of 0.6%, a greater number of such particles is observed, directly demonstrating that higher initiator content improves polymerization efficiency. These particles primarily result from micro-pores formed on the substrate surface due to cement hydration. When the polyacrylate mortar bonds with the substrate, the good fluidity of the acrylate monomers allows partial penetration into the substrate surface. With higher initiator content, the polymerization efficiency increases, leading to a greater degree of crosslinking of the polyacrylate at the bonding interface compared with lower initiator content. Consequently, after polymerization, the interfacial region exhibits a higher density of polyacrylate distribution.

It should be noted that this study only conducted SEM observations on two initiator contents. While these results reflect the main trends, a more comprehensive microstructure–performance relationship requires systematic investigation of additional samples in future studies.

These observations confirm at the microstructural level that initiator content affects the formation of the polymer network, thereby determining the macroscopic mechanical properties of the material. A dense and continuous polymer network structure is the key factor in achieving high-performance polyacrylate mortar.

## 4. Conclusions

This study investigates the effects of initiator content (0.1–1.0%) and polymerization temperature (−20–60 °C) on the mechanical and bonding properties of polyacrylate mortar. Based on experimental measurements, the following conclusions can be drawn:

(1) At 60 °C, the mechanical strength of polyacrylate mortar increases rapidly within 3–5 h and then stabilizes, indicating that the polymerization reaction is essentially complete. To ensure sufficient reaction under all temperature conditions, 12 h was determined as the standard curing time in this study.
(2) There exists an optimal initiator content of 0.6%. When the content is below this value, compressive, flexural, tensile, and bonding strengths increase significantly with higher content. Beyond this threshold, performance growth enters a plateau stage. This indicates that 0.6% is the optimal dosage for achieving efficient monomer conversion and the formation of a complete polymer network in this system.
(3) All mechanical properties show a negative correlation with temperature. From a pure performance perspective, curing at −20 °C results in the highest mechanical strength. This is attributed to the stable redox initiation reaction of BPO/DMA at low temperatures, which favors the formation of polymers with higher molecular weight and more regular structures. Additionally, the polymer chains remain in a glassy state, exhibiting extremely high modulus.
(4) SEM analysis reveals that at an initiator content of 0.6%, the polymer phase is more continuous and compact and adheres more firmly to the aggregates, which microscopically explains the superiority of its macroscopic performance.

Future Work: This study identifies 0.6% as the key optimized parameter for polyacrylate mortar, providing direct guidance for mix design in engineering applications. In scenarios requiring the highest mechanical performance (e.g., under extreme load-bearing conditions), low-temperature curing may be adopted if environmental or curing conditions permit. It must be emphasized that recommending −20 °C as the optimal curing temperature is based on its ability to yield superior mechanical properties. However, we fully recognize that in practical construction—especially under field conditions—maintaining a curing environment at −20 °C is generally infeasible and economically prohibitive. Therefore, from an engineering practicality and cost-effectiveness perspective, curing at ambient temperatures (20–40 °C) is a more realistic and recommended option. Although room-temperature curing results in slightly lower strength compared to the theoretical peak at −20 °C, its performance is still sufficient to meet the requirements of most applications.

The clear trends and optimal ranges identified in this study establish a solid basis for future work. A logical next step would be to employ a Response Surface Methodology (RSM) to develop a quantitative predictive model for the mechanical properties, enabling precise optimization of the formulation and curing conditions for specific application requirements.

## Figures and Tables

**Table 1:** Physical properties of BMA, SF, BPO and DMA.

Chemical Composition: %	BMA	SF	BPO	DMA
Silicon dioxide (SiO_2_)	-	87.29	-	-
Aluminum oxide (Al_2_O_3_)	-	0.47	-	-
Iron (III) oxide (Fe_2_O_3_)	-	0.63	-	-
Calcium oxide (CaO)	-	0.81	-	-
Magnesium oxide (MgO)	-	4.47	-	-
Titanium dioxide (TiO_2_)	-	-	-	-
Sulfur trioxide (SO_3_)	-	0.22	-	-
Sodium oxide (Na_2_O)	-	1.25	-	-
Potassium oxide (K_2_O)	-	1.28	--	-
Loss on ignition	-	2.70	-	-
Molecular formula	C_9_H_14_O_2_	-	C_14_H_10_O_4_	C_8_H_11_N
Melting Point (°C)	<−75	-	105	2.5
Boiling point (°C)	160	-	349.7	193.1
Relative density (g/cm^3^)	0.889		1.334	0.96
Purity	>99.5%	-	-	-

**Table 2:** Composition of aggregates.

Constituent Components	Particle Size (mm)	Mass Percentage (%)
Quartz sand A	2.36–4.75	54.3
Quartz sand B	0.25–0.3	32.5
SF	<0.25	13.2

## Data Availability

The original contributions presented in this study are included in the article. Further inquiries can be directed to the corresponding author.

## Supplementary Materials

The following supporting information can be downloaded at: https://www.mdpi.com/article/10.3390/ma18204737/s1, Table S1: Levene’s Test and ANOVA Results.
